# Effect of ArtemiC in patients with COVID‐19: A Phase II prospective study

**DOI:** 10.1111/jcmm.17337

**Published:** 2022-05-19

**Authors:** Elias Hellou, Jameel Mohsin, Ameer Elemy, Fahed Hakim, Mona Mustafa‐Hellou, Shadi Hamoud

**Affiliations:** ^1^ Department of Cardiology E.M.M.S Hospital Nazareth Israel; ^2^ Department of Cardiology Hillel Yaffe Hospital Hadera Israel; ^3^ Rappaport Faculty of Medicine Technion‐Israel Institute of Technology Haifa Israel; ^4^ Victory Department for COVID‐19 Patients E.M.M.S Hospital Nazareth Israel; ^5^ 26731 Azrieli Faculty of Medicine Bar‐Ilan University Zefat Israel; ^6^ 58880 Department of Internal Medicine E Rambam Health Care Campus Haifa Israel

**Keywords:** ArtemiC, artemisinin, COVID‐19, curcumin, Vitamin C

## Abstract

Despite intensive efforts, there is no effective remedy for COVID‐19. Moreover, vaccination efficacy declines over time and may be compromised against new SARS‐CoV‐2 lineages. Therefore, there remains an unmet need for simple, accessible, low‐cost and effective pharmacological anti‐SARS‐CoV‐2 agents. ArtemiC is a medical product comprising artemisinin, curcumin, frankincense and vitamin C, all of which possess anti‐inflammatory and anti‐oxidant properties. The present Phase II placebo‐controlled, double‐blinded, multi‐centred, prospective study evaluated the efficacy and safety of ArtemiC in patients with COVID‐19. The study included 50 hospitalized symptomatic COVID‐19 patients randomized (2:1) to receive ArtemiC or placebo oral spray, twice daily on Days 1 and 2, beside standard care. A physical examination was performed, and vital signs and blood tests were monitored daily until hospital discharge (or Day 15). A PCR assessment of SARS‐CoV‐2 carriage was performed at screening and on last visit. ArtemiC improved NEWS2 in 91% of patients and shortened durations of abnormal SpO_2_ levels, oxygen supplementation and fever. No treatment‐related adverse events were reported. These findings suggest that ArtemiC curbed deterioration, possibly by limiting cytokine storm of COVID‐19, thus bearing great promise for COVID‐19 patients, particularly those with comorbidities.

## 1 INTRODUCTION

The coronavirus disease 2019 (COVID‐19) pandemic, which initially emerged in Wuhan‐South‐eastern China in 2019, is caused by severe acute respiratory syndrome coronavirus 2 (SARS‐CoV‐2) and is associated with significant morbidity and mortality among vulnerable patients.^1^ This grim situation is mainly attributed to the poor understanding of the pathogenesis of SARS‐CoV‐2‐induced injury to vital organs, particularly in aged patients with diabetes, obesity, hypertension, heart failure and respiratory diseases.^2^, ^3^ Critically ill cases are characterized by acute respiratory distress syndrome (ARDS) and septic shock, as well as multiple organ dysfunction or failure.^2^, ^3^, ^4^ Human angiotensin‐converting enzyme 2 (ACE2) receptor serves as the binding domain of SARS‐CoV‐2 in human host cells, exploiting its high affinity to this enzyme to inflict remarkable damage to key target organs.^5^, ^6^, ^7^

ACE2 is highly expressed in the intestine, heart, kidney, lung and endothelium, where it cleaves angiotensin (Ang) I into Ang 1–9, which, in turn, is converted to Ang 1–7 by ACE2.^8^, ^9^ In addition, ACE2 generates Ang 1–7 directly from Ang II.^9^ Interestingly, Ang II and Ang 1–7 exert opposing physiologic effects on the regulation of microcirculation and inflammation.^9^, ^10^ Specifically, while Ang II induces vasoconstriction, oxidative stress, inflammation, fibrosis and thrombosis, Ang 1–7 provokes, via its receptor, MasR, beneficial actions, including vasodilation, and anti‐inflammatory, anti‐fibrotic, anti‐thrombotic and diuretic/natriuretic effects.^9^, ^10^ This might be of particular relevance to patients with heart failure, diabetes, pulmonary diseases and hypertension, that is clinical settings that are characterized by intense upregulation of ACE2.^1^ The binding of SARS‐CoV‐2 to ACE2 and its subsequent internalization, depletes the cells of this enzyme and of Ang 1–7, conceivably contributing to the devastating cytokine storm characteristic of this disorder.

Thus far, there is no targeted effective pharmacological remedy for COVID‐19. The therapeutic impact of pharmacological interventions such as hydroxychloroquine and Remdesivir are not evidence‐based, and they are administered to critically ill patients with the lack of other valid therapeutic options.^11^, ^12^ Several studies support the use of short‐term, low‐dose corticosteroids in severely ill COVID‐19 patients.^13^ In this context, a comprehensive study demonstrated that the use of dexamethasone in hospitalized patients with COVID‐19 resulted in a lower 28‐day mortality rate among those who were receiving either invasive mechanical ventilation or oxygen alone, but not among those who received no respiratory support.^14^ In line with these findings, medical centres adopted administration of 6 mg dexamethasone daily, for up to 10 days, as a routine therapeutic protocol for patients with severe COVID‐19.^15^ Likewise, the multi‐centre CITRIS‐ALI trial showed that intravenous administration of a moderate dose of ascorbic acid (vitamin C) was safe and reduced mortality among septic patients.^16^ Interestingly, high‐dose vitamin C infusion to patients with COVID‐19 is therapeutically considered for COVID, although whether ascorbic acid can improve the prognosis of these patients is yet to be determined.^17^

The development and approval of effective vaccinations or neutralizing antibodies concentrates may take up to years to reach developing countries, and there is no guarantee that they will be effective against evolving new SARS‐CoV‐2 lineages.^18^, ^19^ In addition, populations that are not eligible for vaccination, namely, paediatric populations,^20^ vaccine hesitancy and unacceptance trend,^21^, ^22^ and populations with low vaccine efficacy, such as haemodialysis patients, and solid organ transplant recipients,^23^, ^24^, ^25^ these barriers may be overcome by a simple, accessible, low‐cost and effective intervention against all SARS viruses, that might restore the pulmonary and microcirculatory malfunction.

The current study examined the safety and tolerability of ArtemiC oral spray in hospitalized COVID‐19 patients, as well as its efficacy in improving major symptoms of these patients. ArtemiC is comprised of artemisinin (6 mg/ml), curcumin (20 mg/ml), frankincense (15 mg/ml) and Vitamin C (60 mg/ml).

## 2 METHODS

In this Phase II, randomized, placebo‐controlled, double‐blinded, multi‐centred, prospective study, 50 adult patients with confirmed SARS‐CoV‐2 infection and hospitalized due to COVID‐19 symptoms, received either ArtemiC or placebo oral spray (2:1) twice a day, beside standard of care (SOC) therapy.

Inclusion criteria were confirmed SARS‐CoV‐2 infection (see Table 1), ≥18 years of age, hospitalized with COVID‐19 symptoms of moderate stable or worsening severity not requiring intensive care unit (ICU) admission, but failing to respond to ongoing standard care, and ability to receive treatment by spray into the oral cavity. Patients on tube feeding or parenteral nutrition, in need of oxygen supply beyond use of nozzles or simple mask as per score 4 (Ordinal Scale for Clinical Improvement Score >4), with respiratory decompensation requiring mechanical ventilation, uncontrolled diabetes mellitus type 2, known autoimmune disease, pregnant or lactating, requiring admission to ICU in the course of the hospitalization at any time prior to completion of the recruitment to the study, or with any condition which, in the opinion of the principal investigator, would prevent full participation in this trial or would interfere with the evaluation of the trial endpoints, were not eligible to participate in the study.

**TABLE 1:** Inclusion and exclusion criteria

Inclusion criteria:
≥18 years old
Hospitalized patients with COVID‐19 of moderate stable or worsening severity not requiring intensive care unit (ICU) admission, who were not experiencing clinical improvement under ongoing standard care.
Subjects under observation or admitted to a controlled facility or hospital (home quarantine was not sufficient)
Patients able to receive treatment by spray into the oral cavity.
Exclusion criteria:
Tube feeding or parenteral nutrition
The need of oxygen supply beyond use of nozzles or simple mask as per score 4 (Ordinal Scale for Clinical Improvement Score >4)
Respiratory decompensation requiring mechanical ventilation
Uncontrolled diabetes mellitus type 2
Known autoimmune disease
Pregnant or lactating women
Need for admission to ICU during the present hospitalization at any time prior to completion of the recruitment to the study
Any condition that would prevent full participation in trial or would interfere with the evaluation of the trial endpoints

Patients were monitored until day 15 or discharge from the hospital. Monitoring included daily physical examination, vital signs (blood pressure, pulse, weight, body temperature) measurements and haematology and biochemistry blood tests such as: complete blood counts, electrolytes, kidney, liver, inflammatory and coagulation indexes. A polymerase chain reaction (PCR)‐based assessment of SARS‐CoV‐2 carriage was performed at screening and on day 15, or at discharge, if it occurred after day 15. Safety was monitored throughout the follow‐up period.

Study products were labelled with randomization numbers at the manufacturing facility and shipped to the study sites. Treatment was administered by the medical staff members in COVID‐19 wards. All patients and researchers were blinded to the administered suspension, that is placebo or drug. Treatment involved administration of 1 ml spray (10 puffs) in the oral cavity, twice a day, at 12‐h intervals on days 1 and 2 of the study, as an add‐on treatment to SOC.

ArtemiC oral spray was given during the treatment session where, subjects received 1 ml oral spray (10 puffs), amounting to a total daily dose of 12 mg artemisinin, 40 mg curcumin, 30 mg frankincense and 120 mg vitamin C. The placebo oral spray is comprised of the same solvent (water), with no active ingredients. Both the active and control sprays were provided in a bottle containing 10 ml spray, which were stored at room temperature (see Table S1 for details concerning the drug preparation and ingredients).

Time to clinical improvement was defined as a ≤2 National Early Warning Score 2 (NEWS2) maintained for at least 24 h. Time to clinical improvement in the active arm was compared to that of the control arm. Patient NEWS2 scaling is amended in supplementary file (Table S2).^26^ The trial was registered in the NIH, ClinicalTrials.gov Identifier: NCT04382040.

### 2.1 Statistical analyses

Baseline characteristics are presented as means and standard deviations for continuous variables and as frequencies and percentages for categorical variables. The primary safety endpoints were assessed by descriptive statistics of adverse events (AEs) and laboratory tests. The incidence of reported AEs and the values of laboratory tests from all subjects are presented with and without regard to relationship to treatment, as determined by the Investigator. Fisher's exact test were applied to assess the inter‐cohort difference in per cent of subjects reporting adverse events. Paired *T*‐test was applied to assess the changes from baseline in laboratory biochemical and haematological values within each study group. ANOVA was applied to assess the statistical significance of the difference in the changes in laboratory results between the study groups. ANOVA was also applied to assess the statistical significance of the difference in the secondary endpoints, including SARS‐CoV‐2 load, respiratory rate, O_2_ saturation, temperature, heart rate and blood pressure, between the study groups. Changes in efficacy parameters were assessed over time for each individual subject. All tests were two‐tailed, and a *p*‐value of ≤0.05 was considered statistically significant. Data were analysed with IBM SPSS statistics software version 27.0. (SPSS Inc.).

## 3 RESULTS

Fifty patients with COVID‐19 who were hospitalized in non‐ICU wards were enrolled in the study; active treatment was administered to 33 patients and placebo to 17 patients. The demographic and baseline characteristics were similar across the two groups, as shown in Table 2. Mean age was approximately 52 years in both cohorts and there was an almost equal percentage of males and females in each cohort. Baseline clinical presentations were similar across the two cohorts, with the majority (~60%) of patients presenting with a NEWS2 of 0 or 1. At baseline, 12.1% of the ArtemiC‐treated patients and 17.6% of the placebo‐treated patients were on supplemental oxygen (*p *= 0.677).

**TABLE 2:** Demographics and baseline characteristics

Population	Treatment Group		*p*‐value
	Active (*N* = 33)	Placebo (*N* = 17)	
Age (years) [Mean ± SD]	52 ± 14	53 ± 14	0.857
Sex, Males (%)	17 (52)	8 (47)	0.708
Race, *n* (%)			0.139
Asian	9 (27)	1 (6)	
White	23 (70)	16 (94)	
African	1 (3)	‐	
Smoker, *n* (%)			0.277
Current	5 (15)	3 (18)	
Past	1 (3)	3 (18)	
Never	26 (79)	11 (65)	
Alcohol consumer, *n* (%)			0.321
Occasional	2 (6)	0	
Weekly	0 (0)	1 (6)	
Never	30 (91)	16 (94)	
NEWS2 [Mean ± SD]	1.5 ± 2.0	1.9 ± 2.1	0.546
NEWS2, *n* (%)			
0	15 (45.4)	4 (23.5)	
1	7 (21.2)	6 (35.3)	
2	3 (9.0)	3 (17.6)	
3	2 (6.0)	1 (5.9)	
4	2 (6.0)	1 (5.9)	
5	2 (6.0)	‐	
6	1 (3.0)	1 (5.9)	
7	1 (3.0)	1 (5.9)	
Supplemental O_2_, *n* (%)	4 (12.1)	3 (17.6)	0.677
Systolic blood pressure (mmHg) [Mean ± SD]	125 ± 18	128 ± 24	0.558
Pulse, (mmHg) [Mean ± SD]	78 ± 13	73 ± 14	0.234
Temperature, °C [Mean ± SD]	36.9 ± 0.5	36.8 ± 0.5	0.783
Minimum, Maximum	36.0, 39.4	36.0, 37.9	
Saturation, % [Mean ± SD]	96.6 ± 2.1	94.9 ± 4.5	0.070

### 3.1 Primary efficacy endpoint

**FIGURE 1:**
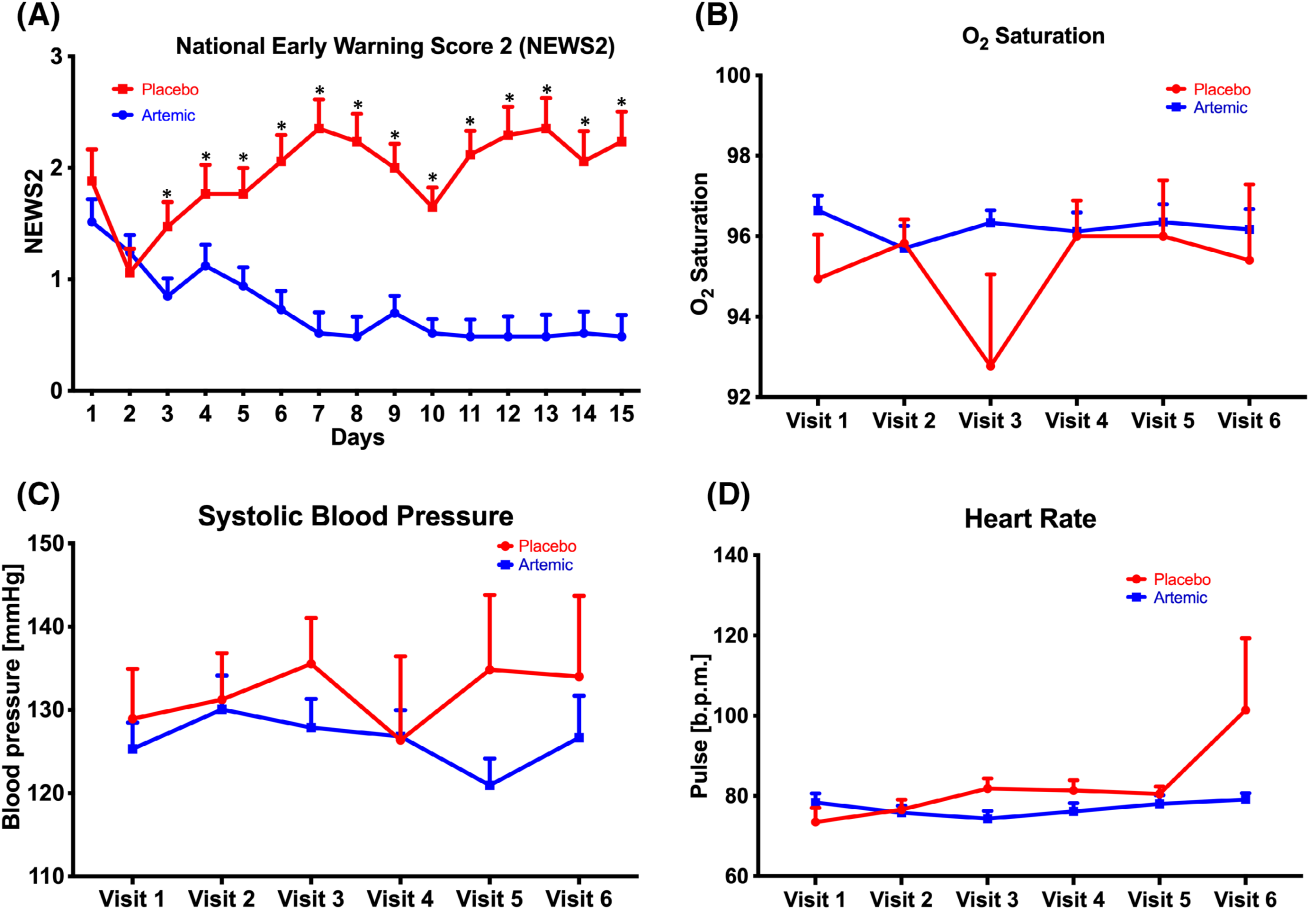
Clinical measures in ArtemiC‐ versus placebo‐treated COVID‐19 patients over time. Treatment of ArtemiC resulted in significantly lower NEWS2 score compared to placebo, as early as Day 3, and difference was consistent up to the end of the study (*p *= 0.042) (A). Treatment with ArtemiC did not affect SpO_2_ saturation (B), blood pressure (C) or heart rate (D)

Subjects treated with ArtemiC showed significantly greater clinical improvement by the end of the follow‐up period, with a mean last‐observed NEWS2 score of 0.52 ± 0.67, versus a mean score of 2.23 ± 3.20 among placebo‐treated subjects (*p *= 0.042) (Figure 1). All but three patients receiving active treatment maintained (59.5%) or showed improved (36.4%) last‐observed NEWS2 scores as compared to baseline. Of note, 13/18 (72.2%) of the patients maintaining their baseline NEWS2 had a score of 0.0 at baseline. Ten (30.3%) ArtemiC‐treated patients had a last documented NEWS2 of ≥2‐points lower than their baseline score. In contrast, 6 placebo‐treated patients (35.3%) showed worsened last‐observed NEWS2 as compared to their baseline score. Imputation by last observation carried forward found a significant difference in NEWS2 of the active versus placebo patients over time (*p *≤ 0.04 from Day 11 and on; Figure 1).

### 3.2 Secondary efficacy endpoints

No group differences were noted for mean oxygen saturation throughout the study. However, there were differences in the number of patients showing abnormal levels of SpO_2_ with increasing time from treatment. Specifically, abnormal SpO_2_ levels were documented in 11 (33.3%) ArtemiC‐treated patients and in 7 (41.2%) placebo‐treated patients at baseline (Table 3, Figure 1). After day 9, there were 2 (6%) patients in the active arm with abnormal SpO_2_ levels, as compared with 4 patients (23.5%) in the placebo arm who were still suffering from abnormal SpO_2_ on Day 11, 3 (17.6%) on Days 12 and 13 and 2 (11.8%) on Day 15. In total, 7 (21.2%) of the ArtemiC‐treated patients required supplemental oxygen versus 5 (29.4%) of the placebo‐treated patients required supplemental oxygen (*p *= 0.728). Mean duration of oxygen support was 2.3 ± 1.4 days in the treatment group and 7.6 ± 4.6 days in the control group (*p *= 0.171). While 12.1% of the subjects in the active treatment arm were receiving supplemental oxygen at baseline, all were weaned off of supportive treatment by Day 6. In contrast, aside from the three placebo‐treated subjects receiving supplemental oxygen at baseline, there were two additional subjects requiring such support during the study period, one of whom receiving supplemental oxygen from Day 4 to Day 13. Two placebo‐treated patients suffered from ARDS and required mechanical ventilation. Overall, 4 placebo‐treated subjects were still on oxygen support at the end of the study.

**TABLE 3:** Last‐observed COVID‐19 carriage and clinical measures (ITT population)

Population	Treatment Group		*p*‐value
	Active (*N* = 33)	Placebo (*N* = 17)	
PCR‐COVID‐19 on last visit, *n* (%)			0.773
Positive	14 (42.4)	8 (47.1)	
Negative	17 (51.5)	9 (52.9)	
Cut‐off value (larger than 35 cycles)	2 (6.0)	‐	
Supplemental O_2_, *n* (%)			0.675
During study (between Day 1 and Day 15)	4 (12.1)	5 (29.4)	
End of study visit	0 (0)	4 (23.5)	
Systolic blood pressure, mmHg			0.310
Mean (SD)	128.7 (16.3)	123.2 (21.0)	
Median	123	119	
Min, Max	100, 163	92, 166	
Pulse, mmHg			0.437
Mean (SD)	76.6 (9.5)	79.1 (12.5)	
Median	80	76	
Min, Max	78, 98	64, 113	
Temperature, °C			0.611
Mean (SD)	36.7 (0.2)	36.7 (0.4)	
Median	36.6	36.7	
Min, Max	36.0, 37.2	36.0, 37.6	
Saturation, %			0.885
Mean (SD)	96.6 (1.5)	96.5 (2.8)	
Median	97	95	
Min, Max	93, 100	72, 100	

While no inter‐cohort differences were noted for mean body temperature, abnormal body temperature (>38.0°C or <36.0°C) was not measured after Day 9 (*n* = 1, 3.1%) in the active arm and after Day 12 in the placebo arm (*n* = 1, 5.9%).

In both treatment arms, all subjects showed within‐range pulse and blood pressure levels by the end of the study. All ArtemiC‐treated patients remained alert throughout the study period (NEWS2 subscale score: 0), whereas two placebo‐treated patients suffered from new‐onset disorientation during the study, which persisted for two days in one patient and 9 days in the other. It should be emphasized that the observed disorientation could be attributed either to the viral infection; however, we could not exclude the pulmonary contribution to this phenomenon. By end of study, the majority of ArtemiC‐treated and placebo‐treated patients (~50%) had a negative PCR test, with no detectable viral traces. Average in‐hospital stay was slightly shorter for patients receiving ArtemiC treatment (7.8 ± 7.3 days) as compared to those treated with placebo (9.0 ± 8.0 days, *p *= 0.918). There was no statistical difference in patient haematological profiles after treatment as compared to baseline values.

### 3.3 ArtemiC safety

In total, 17 adverse events (AEs) were reported in 9 patients receiving active treatment and 44 events in 7 patients receiving placebo treatment (Table 4). Most events were mild (*n* = 8, 47.1%, and *n* = 19, 43.2%, respectively) or moderate (*n* = 7, 41.2% and *n* = 19, 43.2%, respectively). In addition, 11 serious AEs (SAEs) were reported for 3 (9%) ArtemiC‐treated patients and 3 (18%) placebo‐treated patients (*p *= 0.396); 8 of the SAEs were severe. None of the AEs were related to the investigational product. This notion is based on our knowledge that none of the ArtemiC compound exert adverse effects neither in experimental or in clinical use.^27^ All the AEs and SAEs are listed in Appendix 15.2.5 and Appendix 15.2.6, respectively. Due to the complexity of COVID‐19 manifestations in the patient population, alongside high frequency of comorbidities, it was not surprising that many AEs and SAEs were reported in the short study period.

**TABLE 4:** Brief summary of adverse events (safety population)

	Active (*N* = 33)		Placebo (*N* = 17)	
	Subjects, *n* (%)	Events	Subjects, *n* (%)	Events
Any Treatment‐Emergent Adverse Event (TEAE)	9	17	7	44
Severe TEAEs	1	2	2	6
TEAEs Definitely Related to Study Treatment	0	0	0	0
TEAEs Leading to Early Termination	0	0	0	0
Treatment‐Emergent Serious Adverse Events	3	4	3	7
Deaths	0	0	0	0

One patient died five days after completion of participation in the study, while still in hospital. He was 68‐year‐old male, hospitalized due to generalized weakness, breathing difficulties and fever (38.3°C). SpO_2_ was 95% at room air (RA). Respiratory rate was 18 breaths per minute. The patient had a history of congestive heart failure, cerebrovascular accident, renal failure (creatinine 2.4 mg/dl), hemiparesis, type 2 diabetes mellitus, hypertension, hyperlipidaemia, polyneuropathy and anaemia (haemoglobin 8 g/dl). He recruited to the study one day after being diagnosed with COVID‐19. The patient received four doses of placebo, and deteriorated, reaching severe ARDS with bilateral pleural effusion. Despite mechanical ventilation and full intensive care support, on Day 20 the patient suffered cardiac arrest and died after resuscitation.

ARDS developed in an additional placebo‐treated 51‐year‐old male who required mechanical ventilation. From Day 3, the patient experienced worsening anaemia, periorbital oedema, hyperkalaemia, and mild elevation of liver enzymes. Severe hypoxia and metabolic acidosis along with anuria developed. The patient needed mechanical ventilation and haemodialysis treatment, and later was connected to extracorporeal membrane oxygenation (ECMO) support, and was discharged after 3 months to his home.

## 4 DISCUSSION

This first‐in‐human assessment of ArtemiC oral spray treatment in hospitalized COVID‐19 patients demonstrated its safety and tolerability, as well as its potential efficacy in improving COVID‐19 symptoms. Specifically, administration of ArtemiC was associated with maintained or improved NEWS2 in 91% of the patients and with shortened durations of abnormal SpO_2_ levels, oxygen supplementation and fever. No patients in the active treatment cohort required mechanical ventilation, while two placebo‐treated patients suffered from ARDS and subsequently required mechanical ventilation support. These findings suggest that ArtemiC curbed deterioration, possibly by preventing progression to the cytokine storm of COVID‐19 and, therefore, bears great promise for COVID‐19 patients, particularly in those with comorbidities. Moreover, it may significantly reduce hospital loads, which currently pose critical limitations on patient care and outcomes. Promisingly, ArtemiC oral spray was safe and tolerable in hospitalized COVID‐19 patients and elicited no adverse events at the hepatic, renal and hematologic levels.

Despite great investments, there still is no effective remedy for COVID‐19 and most utilized therapeutic approaches are largely supportive, besides preventive vaccinations. For instance, the applied pharmacological interventions including hydroxychloroquine, Remdesivir or vitamin cocktails did not show a marked impact on the mortality and morbidity of COVID‐19 patients.^28^ In parallel, despite the encouraging breakthroughs in the development and approval of effective vaccinations as preventive option for COVID‐19, their efficacy has been shown to wane as levels of the neutralizing antibodies declined several months after vaccination on one hand and the emergence of SARS‐CoV‐2 lineages that escape the vaccines on the other.^29^, ^30^ Thus, there remains an unmet need for the development of simple novel therapeutic protocols that are also suitable for evolving lineages. ArtemiC may be an excellent candidate for the treatment of moderately ill or deteriorating COVID‐19 patients hospitalized in a non‐intensive care unit. This assumption is derived from the composition of this all‐natural oral spray formulation, which includes the following components: artemisinin, curcumin, Boswellia serrata [Indian frankincense] and vitamin C, encapsulated in micelles designed to optimize targeted delivery of lipophilic substances poorly absorbed in the body. The active ingredients have well‐established anti‐inflammatory and anti‐microbial activities, with some integrated for centuries in early traditional Chinese medicine and other natural medicine practices.^31^, ^32^, ^33^, ^34^ Encouragingly, our results clearly demonstrated that ArtemiC was associated with maintained or improved NEWS2 in the vast majority of patients (91%) and attenuation of the durations of abnormal SpO_2_ levels, oxygen supplementation and fever. One of the main manifestations of COVID‐19 is fever and desaturation due to systemic inflammation, including pneumonia. The mechanism underlying this phenomenon involves cytokine storm, hypercoagulopathy, oxidative stress and infiltration of various immune cells into the lung parenchyma, eventually leads to reduced SpO_2_.^35^, ^36^, ^37^, ^38^, ^39^, ^40^ Moreover, no patients in the active treatment cohort required mechanical ventilation, while two placebo‐treated patients developed ARDS and subsequently required mechanical ventilation support.

Although the exact mechanisms underlying the beneficial effects of ArtemiC are largely unknown, it is appealing to assume that this cocktail prevents cytokine storm progression, which has been correlated with progression to dyspnoea, respiratory distress or failure, thromboembolic events, multi‐organ failure and even death,^35^, ^36^, ^37^, ^38^, ^39^, ^40^ all hallmark manifestations of COVID‐19. The fact that the virus induces severe diffuse alveolar damage, alongside an exaggerated inflammatory response,^41^, ^42^, ^43^, ^44^ justifies the use of non‐specific immunosuppression.^13^, ^45^ In this context, the beneficial impact of anti‐inflammatory agents in severely ill COVID‐19 patients, provides further evidence of the central role of the cytokine storm in disease pathogenesis.^14^, ^45^ Furthermore, post‐mortem examinations of COVID‐19 patients found a large accumulation of inflammatory cells in the lung tissues.^46^ The lower ARDS rates and improved oxygen saturation profiles in ArtemiC‐treated patients suggest that this cocktail may interfere with the cytokine storm, although further studies assessing cytokines levels are required.

High oxidative stress is an additional complication of COVID‐19 and contributes to the alveolar injury and thrombosis observed in this patient population.^47^, ^48^ In this regard, it was found that thiol levels are low in COVID‐19 patients.^49^ The role of oxidative stress in COVID‐19 pathology is further supported by potential positive impact of oral and intravenous glutathione (GSH) and N‐acetylcysteine (NAC) on the cytokine storm and ARDS in COVID‐19 patients.^50^, ^51^ Likewise, ascorbic acid (vitamin C) and curcumin exert various pharmacological effects including anti‐bacterial, anti‐cancer, anti‐inflammatory, immuno‐modulatory, anti‐oxidant, anti‐fungal, anti‐mutagenic, and anti‐viral activities,^52^ which may have contributed to the obtained beneficial effects of ArtemiC. One of the remarkable effects of vitamin C is its ability to inhibit various forms of T cell apoptosis.^53^ Besides vitamin C, ArtemiC includes curcumin and artemisinin, which, like vitamin C, impart well‐established anti‐inflammatory and anti‐oxidative effects. It is well known that cytokine storm is characterized by oxidative stress along exaggerated coagulation and inflammation.^37^ Therefore, it is reasonable to assume that ArtemiC acts as a new therapeutic agent against the deleterious impact of SARS‐CoV‐2.^32^, ^33^, ^50^, ^52^, ^53^

In summary, our study demonstrated that ArtemiC oral spray as compared to placebo was associated with rapid and significant clinical improvements in hospitalized COVID‐19 patients. The beneficial effects were expressed by maintained or improved NEWS2 in the vast majority of patients, significantly lower mean last‐observed NEWS2 in 30% of patients, fewer patients with out‐of‐range SpO_2_ levels, a smaller percentage of patients requiring supplemental oxygen, shortened supplemental oxygen support, no patients requiring supplemental oxygen after Day 6 and no cases of new‐onset disorientation in the active cohort. Moreover, no serious adverse events were reported, suggesting that ArtemiC could be a therapeutic agent for COVID‐19, especially in light of the short‐term efficacy of vaccines, and its questionable protection against new SARS‐CoV‐2 lineages. All these encouraging therapeutic properties of ArtemiC are unprecedented as most of the applied medications for COVID‐19 treatment, such as steroids and Remdesivir, are non‐specific, with modest efficacy and exert undesirable side effects.^54^, ^55^, ^56^

## 5 CONCLUSION

This is the first clinical study addressing the safety and tolerability of ArtemiC oral spray and its efficacy in improving symptoms in hospitalized COVID‐19 patients. ArtemiC treatment was associated with clinical improvement, improved SpO_2_ levels and shorter duration of fever. These findings suggest that ArtemiC curbed deterioration, possibly by limiting the cytokine storm of COVID‐19, and bears great promise for COVID‐19 patients, particularly in those with comorbidities.

### 5.1 Study limitations

Despite the encouraging results, the current study suffered from some limitations. These included the small cohort sizes, and failure to study possible mechanisms responsible for the beneficial effects of ArtemiC and to measure biomarkers of coagulation and cardiac and renal injuries. In addition, the relative contribution of each active ingredient remains unknown. Future studies are warranted to better understand efficacy and tolerability of the preparation, besides studying mechanisms of action of the products.

## AUTHOR CONTRIBUTIONS

**Elias Hellou** involved in investigation, writing—original draft (lead), review and editing (lead). **Jameel Mohsin, Fahed Hakim and Ameer Elemy** involved in investigation (supporting). **Mona Mustafa‐Hellou** involved in investigation (supporting), writing—review and editing (supporting). **Shadi Hamou** involved in writing—original draft, review and editing (equal).

## CONFLICT OF INTEREST

The authors confirm that there are no conflicts of interest.

## Supporting information

Table S1‐S2

## Data Availability

Data will be submitted upon request.
