# Supplementary material for: Effect of ArtemiC in patients with COVID‐19: A Phase II prospective study
Source: J Cell Mol Med. 2022 May 19;26(11):3281–9. doi: 10.1111/jcmm.17337 (PMC9170814; doi:10.1111/jcmm.17337)
Supplement: Supplementary file 1 — Table S1‐S2 [file JCMM-26-3281-s001.docx]

**SUPPLEMENTARY**

**Table S1: Composition of ArtemiC**

| **Excipient** | **Manufacturer** | **Reference** |
| --- | --- | --- |
| **Distilled water** |  |  |
| **Ascorbic acid** | Molar Chemicals KFT | Ph. Eur. 07/2019:0253 |
| **Sodium benzoate** | FarmaLabor | Ph. Eur. 01/2017:0123 |
| **Potassium sorbate** | Molar Chemicals KFT | Ph. Eur. 01/2017:0618 |
| **Kolliphor® RH 40** | BTC Europe | Ph. Eur. 01/2017:1083 |
| **Kollisolv® PEG 400** | BTC Europe | Ph. Eur. 01/2017:1444 |
| **Kollidon® 12 PF (PVP)** | BTC Europe | Ph. Eur. 07/2017:0685 |
| **Aqua purificata** | Yasenka | Ph. Eur. 04/2018:0008 |

**Table S2: Patient NEWS2 status was defined as:**

| **Patient state** | **Descriptor** | **Score** |
| --- | --- | --- |
| **Uninfected** | No clinical or virological evidence of infection | 0 |
| **Ambulatory** | No limitation of activities | 1 |
|  | Limitation of activities | 2 |
| **Hospitalized mild disease** | Hospitalized, no oxygen therapy | 3 |
|  | Oxygen by mask or nasal prongs | 4 |
| **Hospitalized severe disease** | Noninvasive ventilation or high-flow oxygen | 5 |
|  | Intubation and mechanical ventilation | 6 |
|  | Ventilation + additional organ support – pressors, RRT, ECMO | 7 |
| **Dead** | Death | 8 |
